# Host Physiologic Changes Induced by Influenza A Virus Lead to *Staphylococcus aureus* Biofilm Dispersion and Transition from Asymptomatic Colonization to Invasive Disease

**DOI:** 10.1128/mBio.01235-16

**Published:** 2016-08-09

**Authors:** Ryan M. Reddinger, Nicole R. Luke-Marshall, Anders P. Hakansson, Anthony A. Campagnari

**Affiliations:** a Department of Microbiology and Immunology, University at Buffalo, State University of New York, Buffalo, New York, USA; b Department of Translational Medicine, Lund University, Lund, Skåne, Sweden

## Abstract

*Staphylococcus aureus* is a ubiquitous opportunistic human pathogen and a major health concern worldwide, causing a wide variety of diseases from mild skin infections to systemic disease. *S. aureus* is a major source of severe secondary bacterial pneumonia after influenza A virus infection, which causes widespread morbidity and mortality. While the phenomenon of secondary bacterial pneumonia is well established, the mechanisms behind the transition from asymptomatic colonization to invasive staphylococcal disease following viral infection remains unknown. In this report, we have shown that *S. aureus* biofilms, grown on an upper respiratory epithelial substratum, disperse in response to host physiologic changes related to viral infection, such as febrile range temperatures, exogenous ATP, norepinephrine, and increased glucose. Mice that were colonized with *S. aureus* and subsequently exposed to these physiologic stimuli or influenza A virus coinfection developed pronounced pneumonia. This study provides novel insight into the transition from colonization to invasive disease, providing a better understanding of the events involved in the pathogenesis of secondary staphylococcal pneumonia.

## IMPORTANCE

In this study, we have determined that host physiologic changes related to influenza A virus infection causes *S. aureus* to disperse from a biofilm state. Additionally, we report that these same host physiologic changes promote *S. aureus* dissemination from the nasal tissue to the lungs in an animal model. Furthermore, this study identifies important aspects involved in the transition of *S. aureus* from asymptomatic colonization to pneumonia.

## INTRODUCTION

*Staphylococcus aureus*, a ubiquitous Gram-positive bacterium and opportunistic pathogen, causes a myriad of diseases ranging from minor to severe skin infections, toxic shock syndrome, osteomyelitis, implant-associated infections, and sepsis (1, 2). *S. aureus* is a substantial burden in the health care setting, as it is one of the leading causes of health care-associated infections and has become much more difficult to manage and treat due to the expansion of antibiotic resistance (3–5).

*S. aureus* is also one of the most common causes of secondary bacterial pneumonia in cases of seasonal influenza and especially during influenza pandemics (6–15). Secondary bacterial pneumonia is a serious condition often associated with respiratory virus infections, particularly influenza A virus (16–19). Secondary staphylococcal pneumonia is often very severe, resulting in extensive damage to the respiratory tract, including necrosis of lung tissue, and is closely linked to increased rates of mortality in young children with or without underlying health issues (14, 20–23). Epidemiologic reports show that nasal colonization occurs in approximately 30 to 80% of the overall population and is prevalent among children. This colonization state serves as a reservoir for invasive staphylococcal disease (8, 19, 24–35). In most instances, colonizing bacteria form biofilms on nasal tissue, allowing *S. aureus* to persist by evading host defenses and antibiotic treatment (36).

Several elegant studies have investigated the link between influenza virus infection and staphylococcal pneumonia, and these studies have uncovered many facets of the interplay between the virus, host, and *S. aureus*. Using a mouse model of coinfection, Iverson et al. and Lee et al. have shown that viral infection primes mice for staphylococcal pneumonia and that these mice displayed higher levels of tissue damage, increased inflammation, and immune cell infiltrates, as well as significantly increased rates of mortality over single-agent infections (37, 38). Additionally, Lijek and Weiser have shown that influenza virus infection prior to staphylococcal infection modulates both innate and adaptive immune responses (39). Viral infection appears to modulate type 1 interferon responses, lower interleukin 1β production with implications for interleukin 27, 10, and 17, reducing the phagocytic clearance ability by inhibiting NADPH oxidase, and reducing the production of antimicrobial peptides (40–45). In addition to immune modulation, influenza virus infection has been shown to compromise the epithelial cells of the respiratory tract, leading to dissemination of staphylococci, thereby contributing to systemic infection (46).

While these studies have provided important insight on secondary staphylococcal pneumonia at the site of infection, little is known about the causes of transition from asymptomatic colonization to the invasive disease state. As such, we have investigated several physiologic changes in the host that are directly related to influenza A virus infection. Our results have identified a number of host factors that cause *S. aureus* to disperse from an asymptomatic colonization state in the nasal tissue and disseminate to the lungs, resulting in pneumonia.

## RESULTS

### *S. aureus* forms robust biofilms *in vitro* on fixed H292 cell substrata.

**FIG 1:**
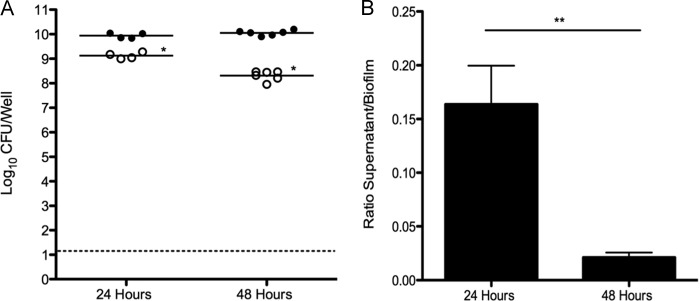
*S. aureus* UAMS-1 forms biofilms *in vitro* on respiratory epithelium substrata. (Left) Biofilm-associated (closed circles) and planktonic (open circles) bacteria were enumerated from individual wells after 24 h (*n* = 4) and 48 h (*n* = 6). Each symbol represents the value for an individual well. The line shows the mean for the wells. The dotted line indicates the limit of CFU that could be detected. (Right) Bacteria were predominately biofilm associated at 48 h. Ratios of supernatant CFU/biofilm CFU were calculated for individual wells. Data presented are representative of three independent replicate experiments. Values that are significantly different are indicated by asterisks as follows: *, *P* = 0.013; **, *P* = 0.0095.

**FIG 2:**
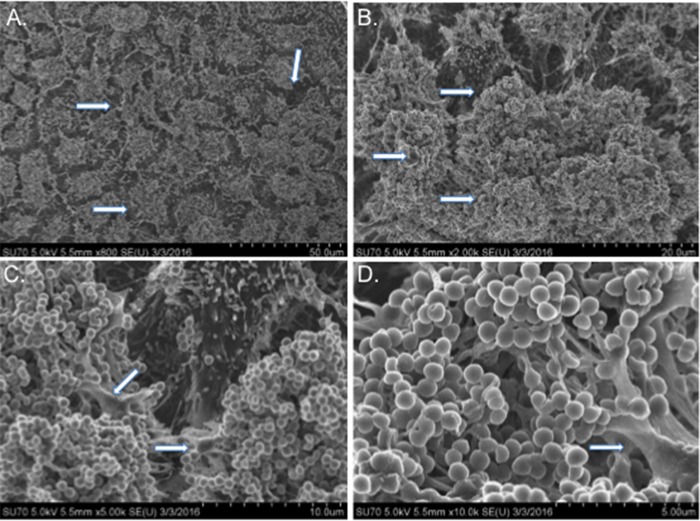
SEM micrographs of *S. aureus* UAMS-1 biofilms grown on H292 cells for 48 h. Arrows indicate water channels (A), tower formations (B), and extracellular matrix (C and D).

Although previous studies have demonstrated that *S. aureus* UAMS-1 forms biofilms on various abiotic surfaces, *S. aureus* most commonly colonizes the human nasal mucosa. Therefore, we performed studies to determine whether UAMS-1 formed biofilms using an *in vitro* model system consisting of prefixed human respiratory epithelial cell substrata. Figure 1 indicates that *S. aureus* UAMS-1 developed biofilms using this system, with greater than 10^10^ CFU per well after 48 h with comparatively less bacteria, approximately 10^8^ CFU in the surrounding supernatant. Biofilms grown for only 24 h had approximately 10-fold-less bacteria in the supernatant compared to biofilm, suggesting that biofilms are not fully formed at this point (Fig. 1). These quantitative data were subsequently supported by scanning electron microscopy (SEM) analysis. Figure 2 clearly shows that strain UAMS-1 formed robust and mature biofilms displaying hallmark characteristics, such as tower formations, water channels, and a well-developed matrix under these more physiologically relevant conditions.

### *S. aureus* responds to host physiologic changes related to virus infection *in vitro*.

**FIG 3:**
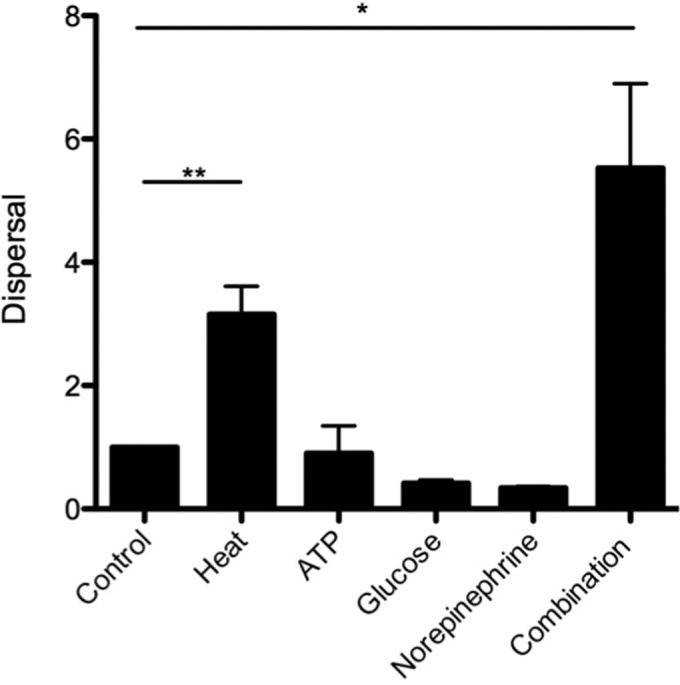
*In vitro* dispersal of 48-h biofilms following 4 h of heat treatment (38.5°C) or exposure to ATP (1 mM), glucose (25 mM), norepinephrine (100 nM), or all four treatments. Dispersal values are presented as the fold change of ratios of supernatant CFU to biofilm CFU values compared to the untreated sample (control). Data presented are cumulative data from four independent replicate experiments. Values that are significantly different are indicated by bars and asterisks as follows: *, *P* = 0.018; **, *P* = 0.007.

Influenza A virus infection leads to a wide variety of physiologic changes in the host due to virus-induced inflammatory responses, including increased body temperature and release of nutrients and cellular “danger signals” associated with extensive tissue damage (47, 48). We investigated whether stimuli related to influenza infection induced *S. aureus* UAMS-1 dispersal from established biofilms. *S. aureus* UAMS-1 responded to increased physiologically relevant temperature by exhibiting approximately fourfold-higher dispersal from the biofilm compared to the control (Fig. 3). Biofilm dispersal due to febrile-range temperatures occurred in clinical isolates NRS8, NRS20, NRS70, NRS100, and NRS123, indicating that this is a species-wide phenomenon and not strain specific (data not shown). Interestingly, exogenous ATP, norepinephrine, and glucose failed to elicit appreciable dispersal on their own, in contrast to data reported for other Gram-positive pathogens (48). Paired combinations of these stimuli also failed to elicit increased dispersal (data not shown). However, when all four of these stimuli were used in combination, *S. aureus* dispersed from the biofilm, suggesting that there was a synergistic effect *in vitro* (Fig. 3). The combination of these conditions is more consistent with a viral infection, as these “danger signals” would be induced simultaneously in the host.

### *S. aureus* strain UAMS-1 colonizes the murine nasal tissue.

**FIG 4:**
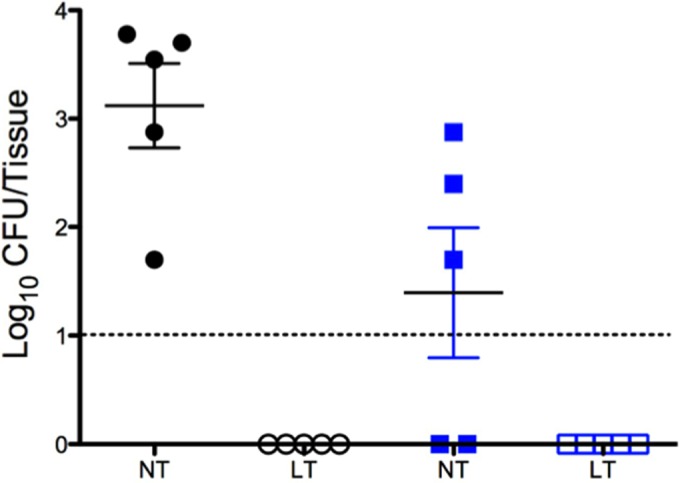
*S. aureus* UAMS-1 colonization of the murine nasal mucosa. The CFU recovered from nasal tissue (NT) and lung tissue (LT) harvested 48 h (black circles) and 7 days (blue squares) after intranasal inoculation. Each symbol represents the CFU recovered from an individual mouse. The dashed line indicates the limit of CFU detection. Data presented are representative of four independent replicate experiments.

Previous coinfection studies with *S. aureus* and influenza A virus involve initial aspiration of the virus followed by aspiration of a large inoculating dose of bacteria 3 to 7 days later (37, 38). While these studies have provided important insight into the pathogenesis of secondary pneumonia, this method circumvents the initial colonizing state that has been shown to be important in disease. Therefore, we established an *in vivo S. aureus* nasal tissue colonization model to study the transition from colonization to invasive disease in the upper respiratory tract. Figure 4 demonstrates that strain UAMS-1 successfully colonizes the murine nasopharynx after intranasal inoculation. The nasal tissues of the challenged mice were stably colonized with approximately 10^3^ CFU per mouse at 48 h with no bacteria detected in the lung tissue (Fig. 4). After 7 days, three of five mice remained colonized with strain UAMS-1 in the nasal tissues, with no UAMS-1 detected in the lungs.

**FIG 5:**
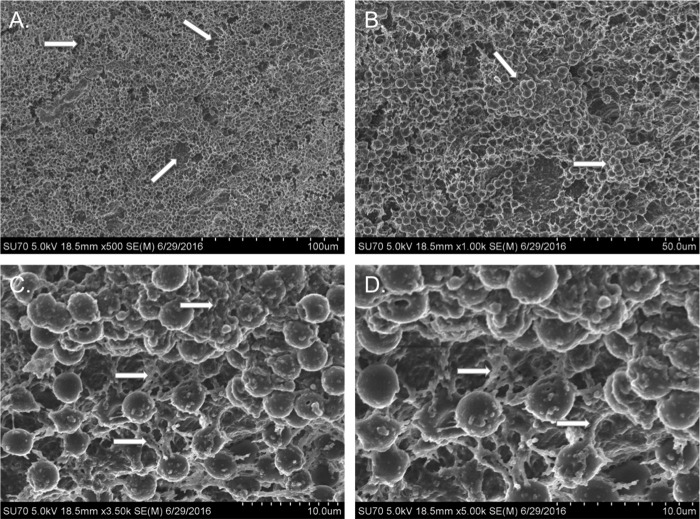
SEM micrographs of nasal-tissue-associated *S. aureus* UAMS-1 biofilms after 48 h of colonization *in vivo*. Arrows indicate water channels (A), tower formations (B), and extracellular matrix (C and D).

These quantitative data were supported by SEM analysis of nasal tissue harvested from mice colonized for 48 h. Figure 5 shows that strain UAMS-1 colonizes the murine nasal tissue in the form of a biofilm with hallmark structures, such as water channels, tower formation, and an extensive extracellular matrix. Taken together, these data indicate that UAMS-1 stably colonizes the murine nasal mucosa for 48 h in biofilms, and in some cases for 1 week, without detectable dissemination into the lungs or development of invasive disease.

### *S. aureus* responds to host physiologic changes induced by virus infection *in vivo*.

**FIG 6:**
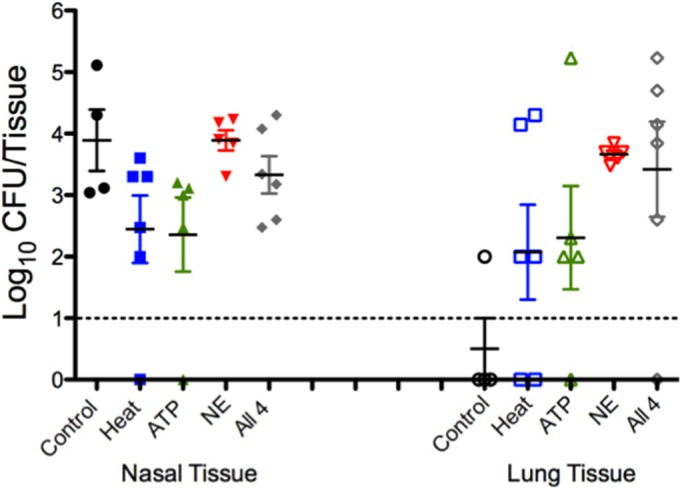
*S. aureus* UAMS-1 dissemination in colonized mice treated with dispersal stimuli. Mice were treated as described in Materials and Methods with heat (blue squares), ATP (green triangles), norepinephrine (NE) (red triangles), or a combination of the stimuli (gray diamonds) or were not challenged or treated (control) (black circles). Each symbol represents the log_10_ CFU/tissue recovered from a tissue sample harvested from an individual mouse (closed symbols, nasal tissue; open symbols, lungs). The dashed line indicates the limit of CFU detection. Data presented are representative of two independent replicate experiments.

Our *in vitro* studies showed that *S. aureus* UAMS-1 dispersed from the biofilm when exposed to host physiologic changes associated with influenza A virus infection. Using our colonization model, we analyzed the effects of virus-induced host factors on strain UAMS-1 *in vivo*. Figure 6 shows that mice exposed to febrile-range temperatures had high levels of UAMS-1 in the lungs. This result supports the conclusion that UAMS-1 disseminates from the nasal tissue and is consistent with our *in vitro* data that UAMS-1 disperses from the biofilm after exposure to febrile-range temperature. However, in contrast to our *in vitro* results, the other host factors also induced dissemination of strain UAMS-1 to the lungs, suggesting that these individual stimuli promote staphylococcal dispersal from nasal biofilms to the lung. Although a strong overall dispersal-response trend was observed *in vivo*, these results were not statistically significant. Taken together, these data suggest that host factors associated with viral infection could stimulate UAMS-1 to leave the stably colonized nasal tissues and invade the lungs after only 4 h of exposure to exogenous stimuli that recapitulates virus-induced host physiologic changes.

### Concomitant influenza A virus infection in asymptomatically colonized mice leads to staphylococcal pneumonia.

**FIG 7:**
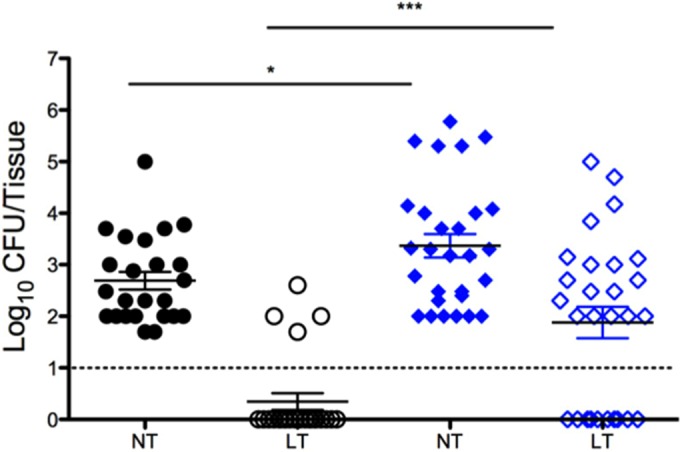
*S. aureus* UAMS-1 dissemination following influenza A virus coinfection. Each symbol represents the CFU recovered from a tissue sample from an individual mouse. The closed points represent nasal tissue (NT), and the open symbols represent lung tissue (LT). The black circles show the results for control mice that received only intranasal bacteria, and the blue diamonds show the results for mice that were colonized and coinfected with influenza virus 48 h later. The dashed line indicates the limit of CFU detection. Data presented are cumulative data from four independent replicate experiments. Values that are significantly different are indicated by bars and asterisks as follows: *, *P* = 0.0325; ***, *P* = 0.0002.

Our results indicated that host factors released in response to viral infection induced bacterial dissemination *in vivo*, thus we performed subsequent studies to determine whether influenza A virus infection elicited secondary staphylococcal pneumonia using our mouse colonization model. Figure 7 shows that after 48 h of colonization, the control group that received *S*. *aureus* UAMS-1 alone had approximately 10^3^ CFU in the nasal tissue. In this experiment, bacteria were detected in the lungs of 4 of the 28 colonized animals (~14%); however, none of these mice exhibited any symptoms of disease, such as piloerection, lethargy, or pyrexia. Mice colonized with strain UAMS-1 for 48 h and subsequently coinfected with influenza A virus show comparable levels of bacteria in nasal tissue; however, these mice also developed a significant secondary bacterial pneumonia with lung bacterial burdens averaging 10^2^ CFU. The coinfected mice showed overt signs of morbidity, including overall lethargy, piloerection, and increased body temperature consistent with the development of invasive disease in murine models compared to control cohorts. UAMS-1 was not recovered from blood samples from either control mice or mice coinfected with influenza A virus. These results indicate that influenza virus infection in mice asymptomatically colonized with UAMS-1 in their nasal tissues induces a transition from colonization to invasive disease and causes pronounced secondary staphylococcal pneumonia.

## DISCUSSION

Staphylococcal pneumonia is often quite severe, resulting in gross tissue damage and necrosis in the lungs, as well as dissemination into the bloodstream, resulting in sepsis. While influenza A virus itself causes tissue damage and disease, the development of secondary bacterial pneumonia is the most significant source of both morbidity and mortality in cases of seasonal influenza and especially during influenza pandemics (6–15). Moreover, epidemiologic studies of the 2009 H1N1 pandemic indicated that secondary bacterial pneumonia with *S. aureus* in particular was a strong indicator of mortality in previously healthy children and patients in intensive care units (13, 14). While there are other etiologic agents of secondary bacterial pneumonia, recent studies have shown concerning increases in the incidence of staphylococcal pneumonia (22, 49–51).

Given the severe nature and increasing prevalence of secondary staphylococcal pneumonia, recent research has focused on discerning the steps involved in pathogenesis. Multiple studies designed to investigate the process of pathogenesis have shown that influenza A virus infection primes mice for staphylococcal pneumonia, often resulting in severe tissue damage and increased mortality, and that the virus modulates the immune systems of infected mice, allowing *S. aureus* to persist in the lungs (40–45, 52). While these studies show aspects of the intricate interplay between these two very different pathogens, directly inoculating bacteria and virus into the lung bypasses the natural route of infection that occurs when colonizing bacteria in the nasal tissues disseminate into the lower respiratory tract. Several studies have shown that colonization precedes invasive disease, implicating this stage as a very important aspect of pathogenesis (35).

We sought to address this gap in knowledge by investigating the transition of *S. aureus* from nasal tissue colonization to invasive disease. While the above-mentioned studies have described the effect of influenza virus infection on modulating the host immune response to allow for *S. aureus* persistence, the effect of “danger signals” produced by viral infection on the bacteria had not been investigated. We established an *in vitro* model using a human respiratory epithelial cell substratum and demonstrated that *S. aureus* forms robust biofilms after a 48-h incubation period. Inclusion of the epithelial substratum and incubation at 34°C more closely mimic the ecological niche of the upper respiratory tract, a common site of *S. aureus* colonization. Biofilms grown in this *in vitro* model displayed characteristic hallmarks of mature staphylococcal biofilms developed *in vivo* on nasal mucosa with organized and defined tower-like structures complete with channels for nutrient exchange and a well-developed biofilm matrix.

Utilizing a physiologically relevant biofilm model system allowed us to investigate the effects of host “danger signals” produced in response to viral infection on *S. aureus* biofilms. Our results showed that *S. aureus* UAMS-1 responds to exogenous stimuli by dispersing from the biofilm. Increased temperature mimicking fever produced a nearly fourfold increase in dispersal, while a combination of four viral-infection-related stimuli (increased temperature, ATP, glucose, and norepinephrine) showed an additive effect, leading to a sixfold increase in dispersal. These results indicate that the physiologic changes that occur in a virally infected host could lead to dispersal from a sessile biofilm community.

These results were further confirmed *in vivo* with our mouse model of nasal colonization. By using intranasal inoculation, we demonstrated that strain UAMS-1 could stably colonize the nasal tissue of mice for 48 h and in some animals up to 7 days without detectable migration to the lungs and subsequent development of invasive disease. However, exposure to the above-mentioned “danger signals” stimulated *S. aureus* to disseminate into the lungs from the nasal tissue and resulted in the development of staphylococcal pneumonia. These results indicate that virus-related physiologic changes serve not only as signaling within the host itself but also across kingdoms to the colonizing bacteria.

While previous studies have demonstrated that viral infections are linked to secondary staphylococcal pneumonia, very little is known about the factors that induce *S. aureus* to transition from colonization to invasive disease. Our novel method of coinfection of the nasal mucosa, the native site of bacterial and viral entry into the host respiratory tract, has provided new insights into possible mechanisms of dissemination. To our knowledge, these are the first studies demonstrating that *S. aureus* can colonize the nasal tissue of mice and subsequently disperse and migrate to the lungs following viral infection leading to secondary staphylococcal pneumonia. These physiologically relevant studies have provided insight into intricate interplay between the host, *S. aureus*, and influenza A virus. While dissemination from the lungs into the bloodstream leading to sepsis is common in the pathogenesis of secondary staphylococcal pneumonia, we were unable to detect strain UAMS-1 in the bloodstream of mice 48 h after virus infection. It may be that in mice, sepsis does not develop until later during infection. Taken together, these data underscore the importance of transition from asymptomatic colonization to invasive disease and highlight the complex nature of cross-kingdom signaling in the development of pneumonia.

Future studies will investigate transcriptional changes that occur in *S. aureus* in response to virally induced host “danger signals” and how these changes are involved in the pathogenesis of secondary staphylococcal pneumonia. Additionally, future studies will investigate whether mice develop sepsis and whether mice succumb to, or clear, these infections.

## MATERIALS AND METHODS

### Ethics statement.

These studies were carried out in accordance with the *Guide for the Care and Use of Laboratory Animals* (53), of the National Institutes of Health and the protocols were approved by the Institutional Animal Care and Use Committee at the University at Buffalo, Buffalo, NY. Experiments were performed under conditions to ensure minimal suffering of the animals involved.

### Reagents.

Cell culture reagents were from Invitrogen, Carlsbad, CA. Bacterial and cell culture materials were from VWR Inc., Radnor, PA. Chemically defined streptococcal growth medium (CDM) was prepared as previously described (54). Sheep blood was purchased from Biolink, Liverpool, NY.

### Cells and bacterial and viral strains.

NCI-H292 bronchial epithelial cells were grown as described previously (55). *Staphylococcus aureus* strain UAMS-1, an osteomyelitis clinical isolate (ATCC 49230) (56), was graciously provided by Steve Gill (University of Rochester, Rochester, NY) for use in these studies. *Staphylococcus aureus* strains NRS8, NRS20, NRS70, NRS100, and NRS123 have been previously described (57) and were used in biofilm formation and dispersal experiments. *S. aureus* was cultured in CDM at 37°C unless otherwise noted. Influenza A virus strain A/PR8/34 (H3N2, ATCC VR-777) was used for the viral coinfection studies.

### Static biofilms on fixed epithelial substratum.

Static staphylococcal biofilms were grown as previously described with slight modifications (58, 59). In brief, 1 × 10^7^ CFU were seeded onto a fixed H292 cell substratum and incubated at 34°C, nasopharyngeal temperature, for 48 h to allow for biofilm development with changes of culture medium at 12-h intervals. A minimum of three independent assays with six replicates each were performed.

### *In vitro* biofilm dispersal.

Biofilm dispersal was performed using previously described virus-induced host response signals that cause *Streptococcus pneumoniae* biofilm dispersal and bacterial release (48). Briefly, static biofilms of *S. aureus* were exposed for 4 h to the following exogenous stimuli: ATP (1 mM), glucose (25 mM), norepinephrine (100 nM), a shift to febrile-range temperature (38.5°C), or all four stimuli together. Supernatants from each biofilm were removed, vortexed, and plated onto blood agar plates for bacterial quantification. The remaining biofilm bacteria were sonicated in phosphate-buffered saline (PBS), vortexed, and plated onto blood agar plates for bacterial quantification. Reported values are the calculated ratio of supernatant to biofilm CFU as a measure of bacterial dispersal.

### Mouse nasal tissue colonization and infection model.

Nasal tissue colonization experiments were performed as previously described with limited modifications (60). Briefly, nonanesthetized 5-week-old BALB/cByJ mice were intranasally inoculated with ~2 × 10^8^ CFU of *S*. *aureus* UAMS-1 in a 20-µl volume by pipetting the inoculum into the nares. For dispersal studies, mice were colonized for 48 h and exposed to the above-mentioned dispersal stimuli for 4 h exactly as described previously (48). Nasal tissue and lungs were collected, homogenized, and dilution plated onto blood agar plates to assess bacterial burden (48, 61, 62). For influenza virus coinfection experiments, mice colonized with strain UAMS-1 for 48 h as indicated above were anesthetized with isofluorane, and 80 PFU of influenza virus A/PR8/34 in 20 µl was pipetted into the nares. Nasal tissue and lungs were collected 48 h later, homogenized, and plated onto blood agar plates to assess bacterial burden. Assays were performed using cohorts of at least three mice per condition on at least two separate occasions.

### Statistical analysis.

The data were analyzed for statistical significance by Mann-Whitney test using Prism 5 software (La Jolla, CA) (38, 46, 63, 64). A *P* value of <0.05 was considered significant.
